# Early Brain Injury: A Common Mechanism in Subarachnoid Hemorrhage and Global Cerebral Ischemia

**DOI:** 10.1155/2013/394036

**Published:** 2013-02-28

**Authors:** Mohammed Sabri, Elliot Lass, R. Loch Macdonald

**Affiliations:** ^1^Division of Neurosurgery, St. Michael's Hospital, 30 Bond Street, Toronto, ON, Canada M5B 1W8; ^2^Labatt Family Centre of Excellence in Brain Injury and Trauma Research, Keenan Research Centre, Li Ka Shing Knowledge Institute of St. Michael's Hospital, 209 Victoria Street, Toronto, ON, Canada M5B 1T8; ^3^Department of Surgery, University of Toronto, 30 Bond Street, Toronto, ON, Canada M5B 1W8

## Abstract

Early brain injury (EBI) has become an area of extreme interest in the recent years and seems to be a common denominator in the pathophysiology of global transient ischemia and subarachnoid hemorrhage (SAH). In this paper, we highlight the importance of cerebral hypoperfusion and other mechanisms that occur in tandem in both pathologies and underline their possible roles in triggering brain injury after hemorrhagic or ischemic strokes.

## 1. Introduction

Aneurysmal subarachnoid hemorrhage (SAH) is associated with significant morbidity and mortality, accounting for up to ~5% of all stroke cases [1, 2]. The mortality from SAH is estimated at 40–45% by 30 days after hemorrhagic onset and up to 15% mortality before hospital admission [3]. After years of research and extensive pathophysiological investigations of SAH, much is known in animal models about pathways that are activated after SAH and that may contribute to brain injury. However, few have proven to be effective therapeutic targets in humans [4, 5]. 

SAH has been suggested in multiple reports to be complex, multisystem, and multifaceted pathogenesis that likely has multiple ongoing processes activated contributing to its final pathogenesis and highly morbid manifestations [4–8]. There are some common effects, however, such as vasoconstriction of both large and small cerebral arteries. As a result, it is difficult to research one pathway, one protein, and one target for potential therapeutic benefits. There has been a shift in research to understand how all the manifestations connect, interact, and further contribute to this pathology. Many strides have been made to understand the common secondary complications that occur after SAH, especially focusing on complications that occur early on, often known as early brain injury (EBI) [9, 10]. Some of the complications that EBI encompasses are delayed neuronal injury/death (DND), oxidative stress and inflammatory destruction of the parenchyma, and ischemic deficits leading to cortical spreading depression (CSD). These complications have been theorized to play a major role in the pathogenesis and may contribute significantly to poor morbidity and outcome after SAH.

Individual studies on several secondary complications have shed light on shared mechanisms and pathways that may be activated after or during or even before the hemorrhage, which may explain a number of these secondary manifestations. Research has also shifted from considering primary angiographic vasospasm as a major contributor to poor outcome to other secondary mechanisms that may also occur early on during the hemorrhage and interact with angiographic vasospasm and predispose the brain to significant delayed injury and poor outcome [10–13].

Recent research has proposed additional mechanisms behind brain predisposition to injury and poor outcome, some of which include global ischemia, delayed cerebral ischemia (DCI), and cortical spreading depression (CSD) [14–16]. Recent work has also focused on trying to delineate the fundamental differences between ischemic deficits and hemorrhagic insult and how early brain injury (EBI) after SAH may be linked to transient global ischemia or may be actually a result of an ischemic deficit introduced early on by the hemorrhage. Does transient global ischemia occur before or during the hemorrhage, and thus predisposing the brain to the secondary complications mentioned? Or is transient global ischemia a separate entity that has its own manifestations, mechanisms, and complications, separate from those pertaining to SAH? 

In this paper we discuss the secondary complications that arise after SAH, its relationship to the pathogenesis, and recent work that has been done to decipher their triggers and roles in poor outcome. Additionally, we will discuss the similarities in pathogenesis between global ischemia and SAH. 

## 2. Global Cerebral Ischemia and Stroke

Ischemia is generally defined as a diminution of cerebral blood flow (CBF) below critical thresholds, resulting in a damage to the entire brain (global ischemia which is necessarily transient if the patient is to survive, and thus it is this type of global ischemia that is often investigated in animal models) or a focal region to which perfusion is relatively low [17, 18]. Global cerebral ischemia occurs when the blood supply to the entire or large part of the brain is impeded [19]. Global cerebral ischemia may also arise from a number of clinical conditions such as cardiac arrest that lasts more than about 10 minutes [19]. This transient insult may result in permanent brain damage and other parenchymal changes that are not completely understood. Since the majority of global cerebral ischemic insults occur due to cardiac arrest, a substantial effort has been allotted to establish protocols for proper management and efficient resuscitation protocols for cardiac arrest patients [19]. Despite optimal resuscitation and adequate ongoing supportive measures, the postarrest period is often accompanied by ongoing cerebral ischemia or no reflow to multiple regions in the brain. This phase of cerebral ischemia is followed by a short phase of cerebral hyperaemia and a prolonged phase of hypoperfusion that lasts from several hours to days and which correlates with significant neurocognitive, behavioural, sensory, and motor deficits [19]. 

Other types of stroke including SAH are associated with a similar pattern of ischemic insult to the brain and may share similarities in cellular pathophysiology. SAH in rats was associated with an upregulation of vasoconstriction-mediating receptors, endothelin B (ET_B_), and serotonin receptors (5-HT_1B_) and with reductions in vasodilators like nitric oxide (NO) [20, 21]. Similarly, in a model of transient global ischemia in rats, Johansson and colleagues demonstrated that animals had prolonged neurological deficits as well as functional upregulation of the same ET_B_ and 5-HT_1B_ receptors in forebrain cerebral arteries. These findings suggest the contribution of cerebral artery vasoconstriction, cerebral hypoperfusion, and neuronal damage to transient global ischemia, which mimics similar findings in SAH [22]. 

## 3. Secondary Complications after SAH

### 3.1. Early Brain Injury: Delayed Neuronal Injury

Cells die after stroke primarily by apoptosis or necrosis [23]. Both are thought to occur after global cerebral ischemia and SAH. The exact pathways activated in these types of stroke are not entirely worked out and there may be different contributions of apoptotic and necrotic cell death. It is documented that transient global cerebral ischemia can trigger multiple cellular events and activate pathways which lead to both apoptotic and necrotic cell death in endothelial, glial, and neuronal cells [24].

During aneurismal rupture causing SAH, the intracranial pressure can increase enough to cause global cerebral ischemia. In some cases, if the bleeding continues and the intracranial pressure does not decrease, then the patient dies immediately, probably secondary to acute cardiac changes secondary to the increased intracranial pressure and near-instantaneous brain death. In survivors, however, the contribution of transient global ischemia to brain injury is variable. Some patients have very small hemorrhages, do not loose consciousness, and thus do not have transient global ischemia. They are still at risk for DCI [25]. Interestingly, patients who become transiently unconscious at the time of their SAH and then awaken have likely had a transient global cerebral ischemic event and may have been at a higher risk of developing DCI [26]. Patients also only develop acute focal cerebral ischemia immediately after SAH in about 3% of cases [27].

Cellular apoptosis is reported to be a mechanism of EBI after the SAH and has been investigated in several studies. These studies focused on large cerebral arteries and found endothelial cell apoptosis after SAH [28, 29]. Neuronal apoptosis in the cortex and hippocampus has been detected after SAH in humans [30]. In animal studies, neurons, astrocytes, and oligodendroglia also exhibited apoptosis after SAH [31]. In some studies, there were fewer neurons in the hippocampus and inner cortical layers 5 days after SAH in rats [32].

The pathways involved in apoptosis after SAH have not been widely investigated. The apoptotic pathways include intrinsic (caspase-independent and mitochondrial) and extrinsic (cell-death receptor) pathways [33–35].

Ischemia caused by increased intracranial pressure (ICP) is probably the first process that activates apoptosis. Apoptosis was observed within minutes of SAH in a rat endovascular perforation model of SAH and persisted for at least 24 hours [12, 35]. Ischemia following a SAH causes apoptotic cell death within the brain through several pathways such as induction of heat shock protein 70 (HSP70) [36]. HSP70 is a sensitive biomarker, which is activated diffusely throughout the brain one day after SAH is induced by endovascular perforation in rats. It continues to be activated 5 days after the SAH [36]. Ischemia also is associated with excitotoxic mechanisms that are mediated through the efflux of the amino acid glutamate. Glutamate activates the n-methyl-d-aspartate (NMDA) receptor following ischemia, resulting in an influx of sodium and calcium into neurons and subsequent neuronal death [37]. This mechanism has been suggested to cause neuronal apoptosis *in vitro* and *in vivo* [38].

The death receptor pathway has been implicated in apoptosis after cerebral ischemia and SAH. This pathway is activated by multiple cell membrane receptors, including the tumor necrosis factor receptor (TNFR), Fas, and DR3-5 [6, 29]. The ligands for these receptors include TNF-*α*, TNF-related apoptosis-inducing ligand, and Fas ligand. This pathway is activated by cerebral ischemia [23]. It has been shown that TNF-*α* is upregulated in the endothelium of dog basilar artery after SAH, and the inhibition of this with broad spectrum apoptosis inhibitors prevented vasospasm [39]. The dogs also had improved neurological outcomes [39]. TNF-*α* binding to TNFR activates caspase 8 and in some cases caspase 10. Downstream caspases are then activated, including caspases 3 and 9. Caspase 3 is a common essential component in the apoptotic pathway [39]. Cleaved caspase 3, a component of the intrinsic, caspase-dependent pathway, was detected in hippocampus and cortex after experimental SAH [12, 40]. The mitochondrial apoptotic pathway is likely involved in cerebral ischemia. Akt (protein kinase B) and mitogen-activated protein kinase (MAPK) are protein kinases that, when activated, inhibit apoptosis by interacting with Bax, Bad, glycogen synthase kinase-3, apoptosis signal-regulating kinase 1, and caspase 9. Akt activity is reduced after cerebral ischemia and its prevention reduced ischemic neuronal death [35]. Inhibiting Akt phosphorylation, which activates it, was associated with EBI after experimental SAH, and overexpression of Akt reduced brain injury [34, 41]. The MAPK may also be involved in EBI [35].

Other mechanisms include caspase-independent intrinsic cell death pathway involved mitochondrial apoptosis-inducing factor (AIF), endonuclease G, and Bcl2/adenovirus E1B 19 kDa-interacting protein (BNIP3) [35]. Nuclear translocation of AIF was found after cerebral ischemia, suggesting the activation of this pathway; however, its role in SAH is less well studied [42].

Autophagy is a process where cells form a multimembrane bound structure called the autophagosome, which sequesters cytoplasm and cell organelles in order to degrade them and recycle cytoplasm [43]. It occurs at basal levels in many tissues and is important in development, differentiation, and remodeling of organs and tissues. Autophagy is linked to apoptosis, but it is unclear if it causes cell death or is activated by some apoptotic pathways [44]. Autophagy has been suggested to provide a neuroprotective role in maintaining cellular homeostasis [43]. On the other hand, under certain conditions, it can have deleterious neurodegenerative effects [45]. After experimental SAH, autophagy has been observed in neurons and astrocytes of the basal frontal cortex on electron microscopy and in brain homogenates by an increase in the amount of membrane-bound microtubule-associated protein I light chain 3 [46]. Cathepsin D, an enzyme associated with degradation of damaged proteins and beclin-1, is also associated with autophagy and also is significantly higher after SAH [46]. Beclin-1 is a protein that interacts with Bcl-2 which is integral in the autophagic process [47]. Activation of autophagy with rapamycin reduced brain injury markers after SAH, whereas inhibition of autophagy with 3-methyladenine aggravated brain injury. This suggests that autophagy plays a neuroprotective role following SAH [44, 47]. 

As discussed above, all of the apoptosis pathways are also likely important in global ischemic deficits after ischemic stroke [19] and may indeed account for some of the EBI after SAH. While neurons and other brain cells die by apoptosis after cerebral ischemia, the predominant mechanism of cell death is caused by necrosis, especially in the core of the ischemic brain [24]. Furthermore, activation of the death receptor pathway in apoptotic-deficient situations causes a sort of a combined form of cell death called necroptosis. Reports have demonstrated that neurons in the core tend to demonstrate liquefaction necrosis, while neurons in the penumbra tend to undergo apoptosis [23, 48, 49]. Apoptosis after cerebral ischemia occurs through intrinsic and extrinsic pathways [48, 50]. In the intrinsic pathway, ischemia results in the generation of permeability pores in the inner mitochondrial membrane, which results in the release of a number of proapoptotic factors and ultimately results in deoxyribonucleic acid (DNA) fragmentation and necrosis [51, 52]. The mitochondrial independent pathways after global ischemia tend to activate death receptors such as TNFR and Fas. Caspases also tend to play a major role in apoptotic activation in both cerebral ischemia and SAH [52, 53]. 

## 4. Nitric Oxide and Nitric Oxide Synthases (NOS)

Nitric oxide has a physiological role as a vasodilator and inhibitor of platelet activation and inflammation [54]. Reduction in NO is thought to contribute to angiographic vasospasm after SAH [55, 56] as well as to EBI [57, 58]. Within 10minutes of SAH in rats, there is acute vasoconstriction probably due to scavenging of NO [58]. NO concentrations subsequently increase above basal levels at 24 hours after SAH [59]. Another mechanism by which NO and NO synthases (NOS) can cause angiographic vasospasm and brain injury is by endothelial NOS uncoupling. This was demonstrated in the brain tissue of mice with SAH, in which there also was increased superoxide and nitrotyrosine production, and significant reduction in NO formation due to the dysfunction of eNOS [60]. Thus, while NO from eNOS might cause vasodilatation and reduce angiographic vasospasm and brain injury, under some conditions it could also be detrimental [61].

While most of the studies in SAH rely on pharmacological manipulations, the contribution of NO to ischemic stroke and transient global ischemia has been assessed in genetically manipulated mice [62, 63]. Mice with reduced neuronal or inducible NOS have reduced infarct sizes, whereas those with eNOS reduction have more [63].

## 5. Oxidative Stress

Reactive oxygen and nitrogen (such as peroxynitrite) species are hypothesized to be important in brain injury after cerebral ischemia and SAH. Multiple studies show that there is release of reactive oxygen species (ROS) after experimental and human SAH [64–69]. Reactive oxygen species can exacerbate inflammation and generalized oxidative stress after SAH by increasing lipid peroxidation, causing direct DNA damage and protein oxidation. These processes in turn activate apoptotic signals and inflammatory cascades that further damage the brain [66]. A major source of ROS after SAH is thought to be oxidation reactions catalyzed by the heme groups of hemoglobin that are obviously abundant in the subarachnoid space after SAH [67].

Reactive oxygen species can be generated by NOS isoforms (endothelial, neuronal, and inducible NOS). Multiple reports have demonstrated that under oxidative environments, NOS, particularly eNOS, can contribute to overproduction of peroxynitrite due to the reaction of NO and superoxide anion radicals [70]. Peroxynitrite oxidizes tetrahydrobiopterin, a cofactor for eNOS, and the zinc-thiolate complex in eNOS, which can uncouple eNOS and lead to generation of superoxide anion radicals [70]. Another source of superoxide anion radicals in the cerebral vasculature is the membrane-bound enzyme nicotinamide adenine dinucleotide phosphate (NADPH) oxidase [71]. NADPH oxidase transfer electrons from NADH or NADPH to molecular oxygen through flavins that are present in the protein structure of the enzyme. This also generates superoxide anion radical which seems to be produced continuously at a low level in cerebral arteries. Structurally, NADPH oxidase has both membrane bound and cytosolic subunits. Functionally, it is a constitutively active enzyme that can mediate vasodilatation, for example, in rabbit cerebral arterioles *in vivo* [72]. The role of NADPH oxidase in the pathophysiology of SAH has not been widely investigated. In one study, inhibition of NADPH oxidase with diphenyleneiodonium reduced middle cerebral artery vasospasm after SAH in rats [73]. Vascular production of superoxide anion radical and NADPH oxidase activity were increased 24 hours after SAH in this model, and this was associated with membrane translocation of p47phox, one of the NADPH oxidase subunits.

Another source of oxidative stress after SAH may be xanthine dehydrogenase. This enzyme is found in endothelial cells where it produces uric acid from purines [74]. Ischemia can convert it to xanthine oxidase, which produces uric acid, superoxide anion radical, and hydrogen peroxide. This is involved in the pathophysiology of brain injury after cerebral ischemia. After SAH, Marklund and colleagues found that delayed cerebral ischemia was associated with increased concentrations of hypoxanthine, allantoin, and uric acid in cerebral microdialysis samples, possibly due to xanthine oxidase activity [75]. However, after experimental SAH in dogs, uric acid was increased in the cerebrospinal fluid, and this was inhibited with allopurinol [76]. This did not reduce angiographic vasospasm. 

Reactive oxygen species are also postulated to be important in cerebral ischemia and infarction. Nitric oxide also can be beneficial after ischemia by mediating vasodilation. However, it can also have toxic effects by, for example, inhibiting complexes I and II in the mitochondrial transport chain [19]. Also, as mentioned above, it can react with superoxide anion radical to produce peroxynitrite. Peroxynitrite generation promotes formation of other ROS such as hydroxyl-free radical and nitrogen dioxide. These nitrosylate tyrosine residues in proteins can result in further structural parenchymal damage [77]. NO has also been shown to upregulate the activity of poly (ADP-ribose) polymerase which leads to neuronal death through ATP consumption [77]. Additionally, in cerebral ischemia, constitutive NOS activity from the endothelial and neuronal NOS isoforms can be increased due to activation of various glutamate receptors, resulting in increased intracellular calcium and cytotoxicity [19]. In general, infarct volume and outcomes are worse in mice lacking eNOS, supporting the beneficial role of vascular endothelial NO [62]. On the other hand, genetic deletion of nNOS or iNOS tends to improve outcomes.

## 6. Inflammation

Inflammation is hypothesized to mediate brain injury and angiographic vasospasm after SAH [78, 79]. There is an increase in proinflammatory cytokines, including TNF-*α*, interleukin 1-*β* (IL1-*β*), and IL6 acutely after experimental SAH [80–82]. Additionally, it has been demonstrated that pharmacologic inhibition of TNF-*α* or IL1-*β* attenuated EBI and improved blood-brain barrier (BBB) function after SAH [80, 81]. Another protein involved in proinflammatory cascade activation is NF-*κ*B, a transcription factor in endothelial cells, that becomes phosphorylated resulting in the subsequent inactivation of I*κ*B-*α* [83]. When NF-*κ*B was activated in the arterial wall, there was an increase in TNF-*α*, IL1-*β*, and adhesion molecules. Pyrrolidine dithiocarbamate, an inhibitor of NF-*κ*B, reduced vasospasm and the increase in the inflammatory cytokines and adhesion molecules. Leukocytes play a role in the immune response following SAH through their role in activating cytokines such as endothelin-1, a power vasoconstrictor that becomes elevated in experimental and clinical SAH [84]. In a study of 224 patients with SAH, a leukocyte count of greater than 15 × 10^9^/L was associated with a 3.3-fold increase in the probability of developing angiographic vasospasm [85]. 

Selectins are from a family of cellular adhesion molecules that play a role in the inflammatory response [79]. They are categorized into leukocyte (L) selectin, platelet (P) selectin, and endothelial (E) selectin, which together mediate the capture, rolling, and adhesion of leukocytes in blood vessels [79]. Functionally, E selectin acts through binding to a carbohydrate site on the leukocytes that helps leukocytes target the site of inflammation. An increase in selectins in cerebrospinal fluid of patients with SAH and in animal models of SAH supports their role in the recruiting of leukocytes to cerebral vessels and brain after SAH [86]. Immunohistochemistry of ruptured cerebral aneurysms found increased E selectin in the aneurysm wall, which could also be a contributor [87]. 

Brain damage after transient global ischemia involves similar pathways to those activated after SAH [19]. Cerebral ischemia leads to migration of peripheral neutrophils and monocytes into the brain. Multiple proinflammatory cytokines are released by neurons and glia, leading to increased selectin and adhesion molecules on cerebral blood vessels, similar to what is observed after SAH. Cytokines are also similarly involved in the pathogenesis of brain injury due to ischemia. Interleukin-1 beta (IL-1*β*) has been reported to play a detrimental role in brain injury, while proinflammatory IL-6 and anti-inflammatory cytokine IL-10 have uncertain roles [19]. Additionally, TNF-*α* has been found to either aggravate ischemic brain injury or to promote development of ischemic tolerance [19, 88]. 

## 7. Blood-Brain Barrier Disruption and Brain Edema

Brain edema is a well-documented phenomenon that occurs days after experimental SAH in multiple animal models [6, 89]. Claassen et al. also concluded, based on interpretation of CT scans, that about 10% of patients had global cerebral edema within 24 hours of SAH [90]. Global cerebral edema was an independent risk factor for poor outcome and mortality. Brain edema may develop due to BBB dysfunction, which is also documented after acute experimental SAH [7, 8, 29]. Multiple processes may contribute to BBB breakdown after SAH, including endothelial cell apoptosis [29]. Blood breakdown products such as oxyhemoglobin and oxidative stress caused by hemoglobin can contribute to BBB disruption [91]. Additionally, proinflammatory cytokines like TNF-*α* and thromboxane A_2_ cause endothelial cell apoptosis and contribute to BBB dysfunction [92]. Inflammatory cytokines increase matrix metalloproteinases (MMP) that also disrupt the BBB. Yan et al. reported that inhibition of p53 ameliorated endothelial cell apoptosis and attenuated BBB disruption and brain edema after SAH in rats [93]. 

Accumulating evidence suggests a role for MMP-9 in the early disruption of the BBB after SAH [94]. MMP-9 degrades the extracellular matrix of the cerebral microvessel basal lamina, which includes collagen IV, laminin, fibronectin, and interendothelial tight junction proteins such as zona occludens-1 [95–97]. Basal lamina degradation starts as early as 6 hours and peaks 48 hours after experimental SAH created by endovascular perforation in rats [98]. Similar to after SAH, in after cerebral ischemia there is a release of proinflammatory cytokines like TNF-*α* and IL-1*β* from glia, leading to generation of adhesion molecules in the vasculature which can result in the weakening of the BBB [99, 100]. Thus, BBB disruption occurs after both a SAH and cerebral ischemia and predisposes to fluid/protein extravasation into the interstitial space resulting in cerebral edema.

## 8. Excitotoxic Amino Acids

Excitatory amino acids may play a role in the pathogenesis of SAH. Germanò et al. reported that the NMDA receptor antagonist, felbamate, attenuated BBB disruption 48 hours after SAH [101]. In view of the known action of felbamate, this suggests a role for NMDA receptor activation in BBB disruption after SAH. Additionally, Unterberg et al. found elevated brain glutamate by intracerebral microdialysis in patients with delayed ischemic deficits after SAH in humans [102]. Similar findings are observed in cerebral ischemia, where glutamate and other excitatory amino acids are increased in brain tissue [19]. The glutamate excitotoxicity hypothesis of brain injury after cerebral ischemia may not be proven, but the process likely occurs after SAH, especially in patients who develop focal ischemia due to delayed angiographic vasospasm or other complications or those with reduced cerebral perfusion pressure from brain swelling and edema. In the excitotoxicity hypothesis, there is brain energy depletion, like in the case with hypoxia-ischemia. Glutamate, one of the most abundant excitatory amino acids, is rapidly effluxed into the extracellular compartment due to neuronal depolarization. It activates NMDA receptors which causes increased intracellular calcium and sodium [19]. Increased calcium activates catabolic enzymes and cell death signaling pathways [38]. Blockade or retardation in the reuptake of excitotoxic amino acids like cysteine results in the depletion of antioxidant intracellular glutathione stores, purportedly causing neuronal injury and death [38]. Furthermore, the use of antiexcitotoxic agents such as NMDA and AMPA-R antagonists conferred neuroprotection through the amelioration of glutamate-induced excitotoxicity caused by hypoxic ischemic injury [38]. This success has not been translated into human ischemic stroke, however. These drugs also are not widely tested in clinical SAH, in part because they failed in human ischemic stroke. 

## 9. Summary

**Figure 1:**
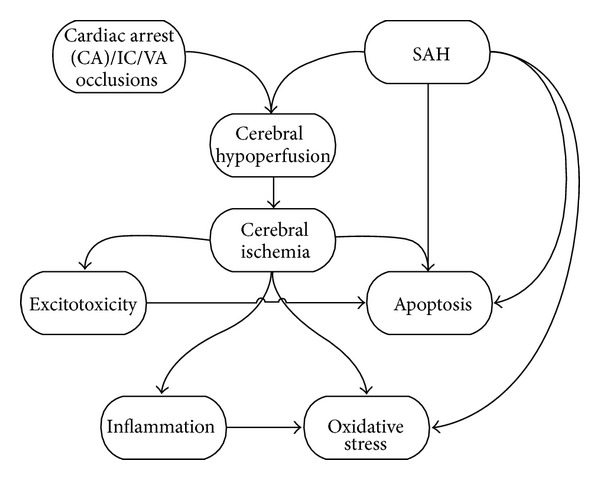
Some hypothetical relationships between cerebral ischemia due to cardiac arrest (CA) and subarachnoid hemorrhage.

The pathophysiology of SAH and cerebral ischemia share some common mechanisms. Cerebral ischemia is often seen as a complication of SAH as well. Early brain injury is also emerging as a key complication and a cause of morbidity and mortality after SAH. Again, common mechanisms may be involved in EBI and cerebral ischemia. Indeed part of EBI may be transient global cerebral ischemia, or at least a common hypoperfusion mechanism that acts between both cerebral insults (Figure 1). Further research is required to help elucidate the differences between EBI in a SAH and ischemic brain injury.
